# Astrocyte‐derived Interleukin‐31 causes poor prognosis in elderly patients with intracerebral hemorrhage

**DOI:** 10.1111/bpa.13245

**Published:** 2024-02-14

**Authors:** Rui Jiang, Zhichao Lu, Chenxing Wang, WenJun Tu, Qi Yao, Jiabing Shen, Xingjia Zhu, Ziheng Wang, Yixun Chen, Yang Yang, Kaijiang Kang, Peipei Gong

**Affiliations:** ^1^ Department of Neurosurgery Affiliated Hospital of Nantong University, Medical School of Nantong University Nantong China; ^2^ Neuro‐Microscopy and Minimally Invasive Translational Medicine Innovation Center Affiliated Hospital of Nantong University Nantong China; ^3^ Jiangsu Medical Innovation Center, Neurological Disease Diagnosis and Treatment Center Affiliated Hospital of Nantong University Nantong China; ^4^ Research Center of Clinical Medicine Affiliated Hospital of Nantong University Nantong China; ^5^ Department of Neurosurgery, Beijing Tiantan Hospital Capital Medical University Beijing China; ^6^ Department of Neurology Affiliated Hospital of Nantong University Nantong China; ^7^ Department of Clinical Bio‐bank Affiliated Hospital of Nantong University Nantong China; ^8^ Centre for Precision Medicine Research and Training, Faculty of Health Sciences University of Macau Taipa China; ^9^ Department of Trauma Center Affiliated Hospital of Nantong University, Medical school of Nantong University Nantong China; ^10^ Department of Neurology, Beijing Tiantan Hospital Capital Medical University Beijing China

**Keywords:** astrocyte, IL31, intracerebral hemorrhage, microglia

## Abstract

The incidence of intracerebral hemorrhage (ICH) is increasing every year, with very high rates of mortality and disability. The prognosis of elderly ICH patients is extremely unfavorable. Interleukin, as an important participant in building the inflammatory microenvironment of the central nervous system after ICH, has long been the focus of neuroimmunology research. However, there are no studies on the role IL31 play in the pathologic process of ICH. We collected para‐lesion tissue for immunofluorescence and flow cytometry from the elderly and young ICH patients who underwent surgery. Here, we found that IL31 expression in the lesion of elderly ICH patients was significantly higher than that of young patients. The activation of astrocytes after ICH releases a large amount of IL31, which binds to microglia through IL31R, causing a large number of microglia to converge to the hematoma area, leading to the spread of neuroinflammation, apoptosis of neurons, and ultimately resulting in poorer recovery of nerve function. Interfering with IL31 expression suppresses neuroinflammation and promotes the recovery of neurological function. Our study demonstrated that elderly patients release more IL31 after ICH than young patients. IL31 promotes the progression of neuroinflammation, leading to neuronal apoptosis as well as neurological decline. Suppression of high IL31 concentrations in the brain after ICH may be a promising therapeutic strategy for ICH.

## Graphical abstract

High concentrations of astrocyte‐derived IL31 in the central nervous system (CNS) immune microenvironment of elderly intracerebral hemorrhage (ICH) patients lead to neurological deficits and poor prognosis. The massive activation of astrocytes after ICH in aged mice releases IL31, which binds to microglia indicating that IL31R binds to each other causing massive chemotaxis of microglia. Subsequent activation of N‐methyl‐D‐aspartic acid receptor (NMDA) receptors, P65 and P53, and so forth, causes degenerative changes in neurons, resulting in neuronal apoptosis and leading to a malignant outcome. In contrast, young mice are more inhibited NMDA receptor, P65 and P53, and so forth, which reduce neuronal apoptosis and thus promotes the recovery of neurological function.

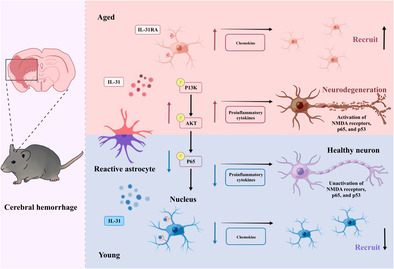

## 1 INTRODUCTION

The incidence of intracerebral hemorrhage (ICH) has been steadily increasing over the years [1, 2]. The high mortality and disability rates among patients with ICH impose a significant burden on families and society, making it a pressing health concern for all residents [3, 4]. Therefore, delving deeper into the pathogenesis of brain injury following ICH can potentially enhance the clinical prognosis of ICH [5]. Currently, it is widely acknowledged that neuroinflammation secondary to ICH significantly influences patient regression and outcomes [6, 7]. Elderly individuals, being more susceptible to ICH, exhibit more intricate and intensified inflammatory responses after central nervous system (CNS) injury, which contributes to the poorer prognosis in this demographic [8]. To bolster the survival rates and promote neurological recovery after ICH, especially in elderly patients, it is imperative to effectively regulate and modulate the CNS immune microenvironment.

Interleukins (ILs), as pivotal contributors to shaping the inflammatory microenvironment of the CNS post‐ICH, have garnered extensive attention in neuroinflammation research [9, 10]. Previous studies have primarily concentrated on elucidating the functions of IL1, IL4, and IL6 families within the CNS [11, 12, 13]. However, ILs serve as a multimember cytokine family. There are still many family members whose functions remain to be explored, such as IL31. IL31 has been associated with various inflammatory skin conditions, including atopic dermatitis [14, 15, 16]. It is typically attributed to Th2 cells, and studies have demonstrated its role in inducing itching, activating immune responses, and influencing the pathology of atopic dermatitis primarily through signaling pathways such as JAK [17, 18]. However, the role of IL31 in the shaping of the immune microenvironment after ICH and its role in the post‐ICH pathologic course remains unclear [19].

To scrutinize the role of IL31 in the pathogenic progression of ICH across various age groups, this study initially selected a cohort comprising elderly and young patients with ICH. During surgery, brain tissues adjacent to the cortical fistulas were procured and subsequently categorized into injury and parainjury areas based on their distance from the hematoma. The expression of IL‐31 was assessed using flow cytometry, polymerase chain reaction (PCR), and immunofluorescence. Subsequently, C57BL/6 mice at 4 and 18 months were chosen to represent the young and elderly stages, respectively, and ICH models were established accordingly. In summary, this study investigated the specific biological functions enacted by IL31 and its potential molecular pathways following ICH, particularly in elderly patients with ICH. This study aimed to bridge existing research gaps in this domain. Simultaneously, the development of monoclonal antibodies targeting this target holds promise for translating laboratory findings into clinical applications, offering substantial clinical therapeutic value.

## 2 MATERIALS AND METHODS

### 2.1 Sample collection and evaluation of patients with ICH

Participants in the study were adults who underwent cortical fistulas to remove hematomas for spontaneous basal ganglia hemorrhage and who provided prior written informed consent to the neurosurgical staff in the presence of a witness. In all cases, consent for the study was obtained before surgery. Pathological specimens were obtained from tissue specimens of temporal lobe dermatostomy. Human samples were gathered by the protocol approved by Nantong University Hospital's Institutional Review Board (2022‐K004). Brain tissue from the cortical fistula of 10 (5 in the young group: 30 ± 5.34 years old and 5 in the elderly group: 75 ± 8.62 years old) patients undergoing ICH surgery was collected for brain tissue flow testing. All 10 patients with ICH underwent hematoma removal within 72 h after the onset of the disease, and fresh brain tissue was collected from the area surrounding the hematoma, close to the hematoma area as the injury area, and close to the cortical portion as the parietal area of injury. The corresponding tissue homogenates were collected and subjected to flow cytometry, immunofluorescence, and PCR.

### 2.2 Experimental animals

C57BL/6 mice, male, both young and elderly (young, 4 months; elderly, 18 months), were obtained from the Nantong University Animal Experiment Center (Nantong, China). All mice were kept pathogen‐free and housed in cages with no more than five animals per cage under standardized light–dark cycle settings with free access to food and drink. The vivarium was kept at a constant temperature (21°C) and humidity (50%–60%). The Nantong University Laboratory Animal Ethics Committee accepted the animal research (S20180816‐012).

### 2.3 Establishment of ICH models

As previously explained, the ICH model was induced. Mice were anesthetized with isoflurane gas and placed on a stereotaxic machine while being anesthetized with 1%–3% isoflurane inhalation and ventilation with oxygen‐enriched air (20% O_2_:80% air) via a nose cone. A total of 20 μL blood was taken from the tail vein as the injection autologous blood, and 15 μL fresh blood was injected into the right striatum (coordinates: 0.5 mm anterior, 4 mm ventral, and 2 mm lateral to the bregma) at a rate of 3 μL/min using a Hamilton syringe (Hamilton, USA). To prevent reflux, the needle was left in place for 10 min. Finally, bone wax was used to seal the skull opening, and the scalp was sutured and sterilized. The mice's body temperature was kept at 37.0 ± 0.5°C throughout the procedure. Sham‐operated animals received a saline injection in the same area and volume as the experimental mice. Except for those who died or had a neurologic deficit assessment score of more than 23 out of 24 within 24 h of surgery, all animals were included in the final analysis.

### 2.4 Immunofluorescence analysis

Mice are anesthetized and pericardially perfused with 0.9% saline and 4% paraformaldehyde 3 days after ICH. The brains were removed, postfixed overnight at room temperature in the same fixative, then immersed in a 20% sucrose solution for 2–3 days, followed by another 3–4 days in a 30% sucrose solution. Using a cryostat, the tissues were sliced into 12 μm slices and stored at −20°C until needed. Our previously described methodology was used to prepare and stain 12 μm thick coronal slices of brain tissue. Frozen sections were incubated using Quick Antigen Retrieval Solution (Solarbio, China) for 2 h at room temperature. Sections were washed three times with phosphate buffer saline (PBS), preincubated with 5% blocking serum for 1 hour, and then incubated with primary antibodies overnight at 4°C. The slices were treated with the secondary antibody mixture for 1 h at 37°C the following day. 4',6‐diamidino‐2‐phenylindole (DAPI) (Solarbio, China) was used to mark cell nuclei. A fluorescent microscope (DM 5000B; Leica, German) was used to analyze the sections. The antibodies used for immunofluorescence are listed in the antibody Materials and Methods section.

### 2.5 Morris water maze

The pool (0.8 m in diameter) was filled with water (60 cm deep, 22–24°C) and painted white to make it opaque. Visual cues were placed on the testing room's wall about 1 m from the pool's edge, and the 10‐by‐10‐centimeter escape platform was buried 2 cm below the surface of the water. Mice were placed in individual cages with plenty of paper towels for the duration of the training. Each of the 4 consecutive training days included eight tries divided into two blocks of 2 min each. A training trial was completed when the mouse ascended and remained on the platform for 2 s or spent 60 s in the pool, with the mouse remaining on the platform for an extra 30 s after each trial. Before returning to their home cage, mice were examined in groups of five. The beginning points were altered every day, but they remained consistent for all mice examined. On Day 5, the platform was removed during the probing trail, and each animal was given 60 s to navigate the pool. The ANY‐maze (Stoelting, USA) was used to track, record, and analyze swimming. Mice that had not received early training were handled in the test room and then returned to their cage to control for the effects of excessive handling.

### 2.6 Rotarod test

An accelerated rotation test was performed on mice before ICH and on Days 1, 7, and 14 after ICH using a rotarod device to assess motor coordination. Mice were placed on an accelerating rotarod apparatus (Ugo Basile, USA) and allowed to move freely as the cylinder accelerated from 5 to 40 revolutions per minute over 5 min. The latency to fall was measured when the mouse fell off the rod or rode the cylinder for two consecutive revolutions without regaining control. Each training day included four trials with a 30‐min recovery in between. Mice that had not received early training were handled in the test room and then returned to their cage to control for the effects of excessive handling.

### 2.7 Intracerebroventricular viral injection

Mice were anesthetized with isoflurane gas and placed on a stereotaxic machine after being anesthetized with 1%–3% isoflurane inhalation and ventilated with oxygen‐enriched air (20% O_2_:80% air) via a nose cone. The adeno‐associated virus vector (Genechem, China) was injected intracerebroventricularly to a depth of 2 mm into the ipsilateral ventricle 1 mm lateral to the superior sagittal sinus, and 2 mm rostral to the transverse sinus. To prevent leakage, the needle remained in the brain for 5 min after injection at a rate of 0.5 μL/min. Sutures were used to repair the drill holes and bone wax was used to close the incisions. Mice were separated into recovery cages. The mice's body temperature was kept at 37.0 ± 0.5°C throughout the procedure.

### 2.8 Flow cytometry

Tissues from mice with injuries, as well as patient specimens, were obtained. The tissues were chopped and treated in collagenase IV at 37°C for 30 min before being rinsed with PBS and myelin debris was removed by centrifugation in 30% percoll (Sigma Aldrich, German), followed by rinsing with PBS and resuspension in 1% BSA. After that, fluorescent‐conjugated antibodies were added to the cell suspension and stained on ice for 30 min. After surface marker labeling, cells were fixed in fixing buffer for 20 min and then washed twice in permeabilization buffer. Following that, antibodies against intracellular molecules were introduced and stained for 45 min. After that, the cells were washed and suspended in flow cytometry buffer.

The essential resources table lists all antibodies used in flow cytometry staining. A FACSAria III flow cytometer was used to collect data (BD, USA), which was then analyzed using the FlowJo software.

### 2.9 Western blot

After perfusing the heart with cold PBS, the brain was carefully removed and immediately placed in an 80°C ultra‐low‐temperature refrigerator. The tissues were then placed in radioimmunoprecipitation assay buffer (RIPA) containing a 1% protease inhibitor cocktail and a 1% phosphatase inhibitor cocktail (Beyotime, China) before being lysed by ultrasound in an ice‐cold environment. After centrifugation, the supernatant was collected, and the protein concentration was detected and analyzed using the bicinchoninic acid (BCA) protein analysis kit. sodium dodecyl sulfate polyacrylamide gel electrophoresis (SDS‐PAGE) on a 10%–12.5% gel separated proteins of varied molecular weights, which were then transferred to a 0.45 μm polyvinylidene fluoride membrane after boiling (Billerica Millipore, USA). After 2 h of blocking with 5% bovine serum albumin, the membranes were treated with the primary antibody overnight. The membranes were incubated with secondary antibodies for 1 h at room temperature the next day, and protein bands were detected using ECL (Billerica Millipore, USA). The Chemidoc detection equipment (Bio‐Rad, USA) and ImageJ software (National Institutes of Health, USA) were used to characterize and quantify protein striping signals. The antibody is described in Table S1.

### 2.10 TUNEL assay

The TUNEL assay was performed using the One‐Step TUNEL Apoptosis Assay Kit (Beyotime, China). The TUNEL reaction mixture (50 μL) was incubated for 60 min at 37°C. Fluorescence microscopy was used to examine cells after they had been cleaned with PBS.

### 2.11 Quantitative reverse transcription PCR

As we previously reported [20, 21], after disrupting and homogenizing the tissue in 1 mL TRIzol (Thermo‐Fisher Scientific, USA), followed by 200 μL chloroform and vortexed for 15 s, RNA was isolated from the brain. To achieve phase separation, the samples were placed at room temperature for 10 min before being centrifuged for 10 min at 12,000 g at 4°C. The top layer (which contained RNA) was transferred to a different tube and precipitated with the same amount of isopropanol. The ethanol was removed from the samples after centrifuging them for 10 min at 8000 g at 4°C, and the samples were air‐dried. The RNA pellet was redissolved in Rnase‐free 20 μL ddH_2_O. NanoDrop system (Thermo‐Fisher Scientific, USA) was used to calculate RNA concentration. For reverse transcription, 0.75 mg RNA (total volume 40 μL diluted in dH_2_O) was incubated for 10 min at 70°C in 5 μL random hexamers. Samples were placed on ice and a master mixture of 0.5 μL reverse transcriptase, 0.5 μL RNase Inhibitor, 2 μL deoxy‐ribonucleoside triphosphate, and 12 μL reverse transcriptase buffer (Thermo‐Fisher Scientific, USA) were added. The samples were incubated at room temperature for 10 min before being placed in a 42°C water bath for 45 min. The samples were incubated for 3 min at 99°C before being placed on ice and kept for future use.

RNA (2 μg) was reverse transcribed in each sample using the TMRT kit (Takara Bio Inc, Japan), and quantitative reverse transcription PCR (qRT‐PCR) was performed on an ABI QuantStudio5 Q5 real‐time PCR System (Thermo‐Fisher Scientific, USA) using SYBR Green (Roche, Germany). To measure and normalize relative mRNA expression to GAPDH expression, the 2‐CT method was utilized. The fold change in mRNA expression was assessed in comparison to the sham‐operated group. The primer sequences used for PCR are provided in Table S2.

### 2.12 Modified neurological severity score for neuro behavioral functioning assessment

As previously reported, the modified neurological severity score (mNSS) was computed. The neurological deficits were assessed by a researcher who was not aware of the experimental groups. To evaluate the neurological function, the mNSS test was performed. On a scale of 0–14 (normal, 0; maximum, 14), the score was assigned. A score of 10–14 indicates severe injury, a score of 5–9 suggests moderate injury, and a score of 1–4 indicates minor injury. In the impairment severity scores, one point is assigned for the inability to conduct a test or the lack of a tested reflex; the higher the score, the more severe the injury.

### 2.13 Drug treatment

PI3K (HY‐12068) and TAK‐1 (HY‐103490) signaling pathway inhibitors were procured from MedChemExpress (MCE, USA), and they were used to observe the prognostic effects in ICH mice after inhibition of the PI3K‐AKT signaling pathway and TAK‐1 signaling pathway.

### 2.14 Statistical analysis

All data were analyzed using GraphPad Prism 9.4.1 and are presented as mean ± standard error of the mean (SEM). The unpaired Student's *t*‐test, one‐way analysis of variance (ANOVA), and two‐way ANOVA were used to compare the results. A *p*‐value x 0.05 was considered statistically significant. Each experiment was repeated at least three times.

## 3 RESULTS

### 3.1 IL‐31 expression is much higher in brain tissues from hematoma areas in elderly people than in young people

Elderly patients with ICH typically experience a less favorable prognosis than their young counterparts. To investigate the underlying reasons for this discrepancy, five young patients (mean age 30 ± 5.34 years) and five elderly patients (mean age 75 ± 8.62 years) with ICH in the basal ganglia region were selected, all of whom underwent surgical treatment. During the surgical procedure, the brain tissue adjacent to the cortical fistula opening was extracted, with the portion closer to the cortex designated as the parainjury area, and the portion nearer to the hematoma identified as the injury area (Figure S1A).

Initially, the variance in cellular composition around the injury site following ICH was observed in both elderly and young patients. The obtained tissues were utilized for flow cytometry. The findings indicated that in elderly patients, the number of astrocytes and microglia around the injury site was significantly higher than that in their young counterparts, whereas the number of neurons was markedly diminished compared with young patients (Figure S1B,C). The activation of astrocytes and microglia often led to heightened inflammation, while neuronal deficits were correlated with poor recovery of neurological function. This observation aligns with the less favorable prognosis seen in elderly patients.

**FIGURE 1:**
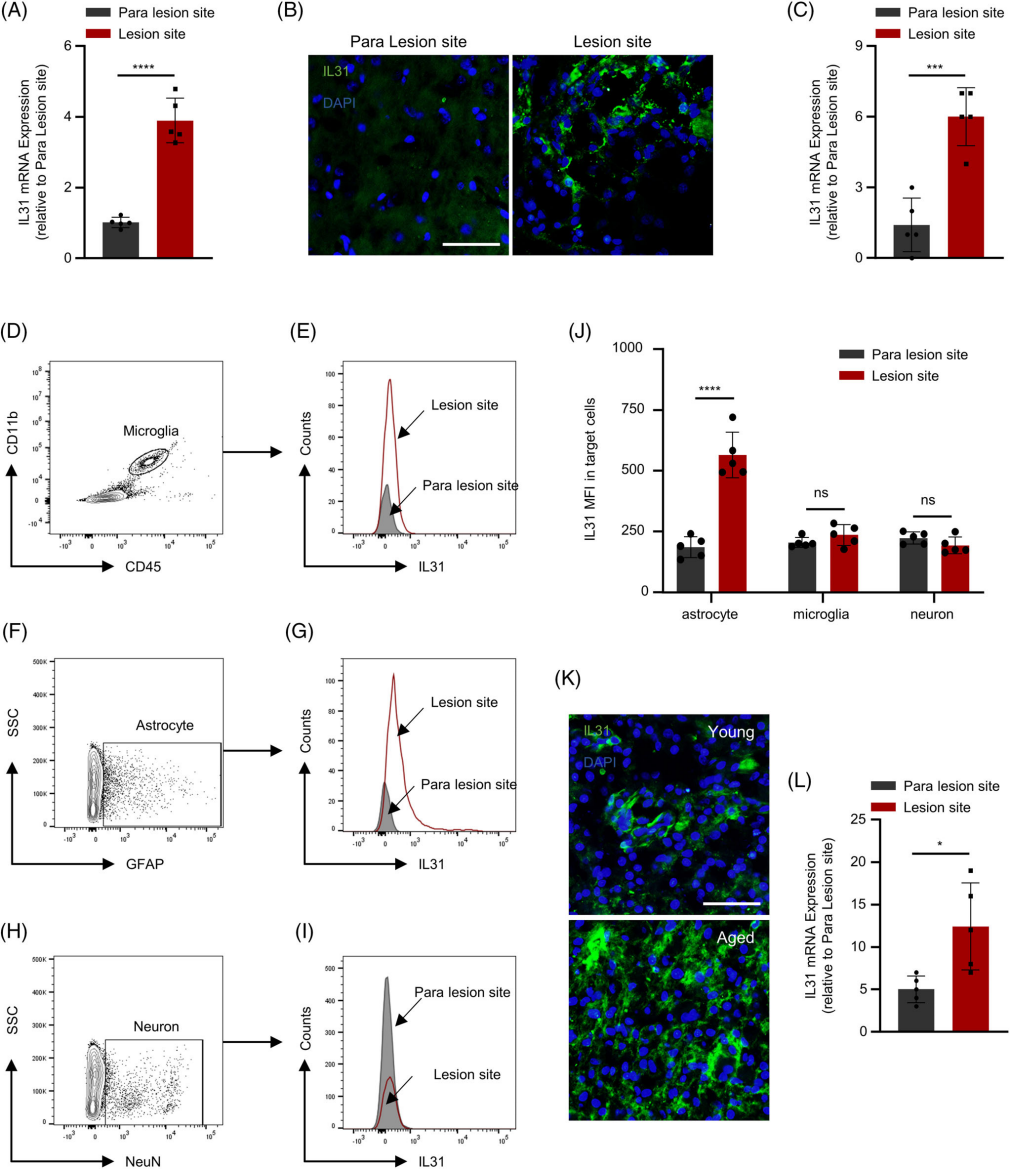
The expression of IL‐31 in the brain tissue of hematoma area of the elderly is much higher than that of young patient. (A) PCR detection of IL31 expression in hematoma area and para‐lesion brain tissue (*n* = 5). (B,C) Immunofluorescence detection of IL31 expression in hematoma area and para‐lesion area and statistics (*n* = 5). (D–J) Flow assay of the ratio of astrocytes, microglia, and neurons in hematoma area and para‐lesion area (*n* = 5). (K) Immunofluorescence detection of IL31 expression in hematoma area of  young and elderly patients and statistics (*n* = 5; *p < 0.05; **p < 0.01; ***p < 0.001; ****p < 0.0001).

IL31, a crucial member of the IL family, was further investigated to elucidate its role post‐ICH. Initially, the alterations in IL31 expression were examined across different regions. PCR results indicated a substantial upregulation of IL31 mRNA expression in the injury region proximate to the hematoma, in contrast to the injury parietal region near the cortex (Figure 1A). This was corroborated by immunofluorescence assay results from brain tissue sections: proximity to the hematoma correlated with the higher fluorescence expression intensity of IL31 (Figure 1B,C). This strongly suggests a notable surge in IL31 expression following ICH.

To pinpoint the source of IL31 after ICH, this study assessed its expression in astrocytes, microglia, and neurons across various regions through the flow cytometry assay. The findings revealed a notably higher expression of IL31 in astrocytes within the injury area than within the parainjury area (Figure 1D,E). Conversely, IL31 expression in microglia and neurons showed no significant change (Figure 1F–J). This implies that post‐ICH, activated astrocytes release substantial amounts of IL31, triggering the escalation of inflammation and the deterioration of neurological function.

We then detected IL31 expression in tissues at the site of injury in elderly and young patients using an immunofluorescence assay. This analysis revealed a significantly higher expression of IL31 in the tissues of elderly patients (Figure 1K,L). These results strongly suggest a notable increase in IL31 expression after ICH in elderly patients, potentially serving as a pivotal factor contributing to the less favorable prognosis.

### 3.2 Elderly mice express higher IL31 and cause the spread of inflammation after ICH

**FIGURE 2:**
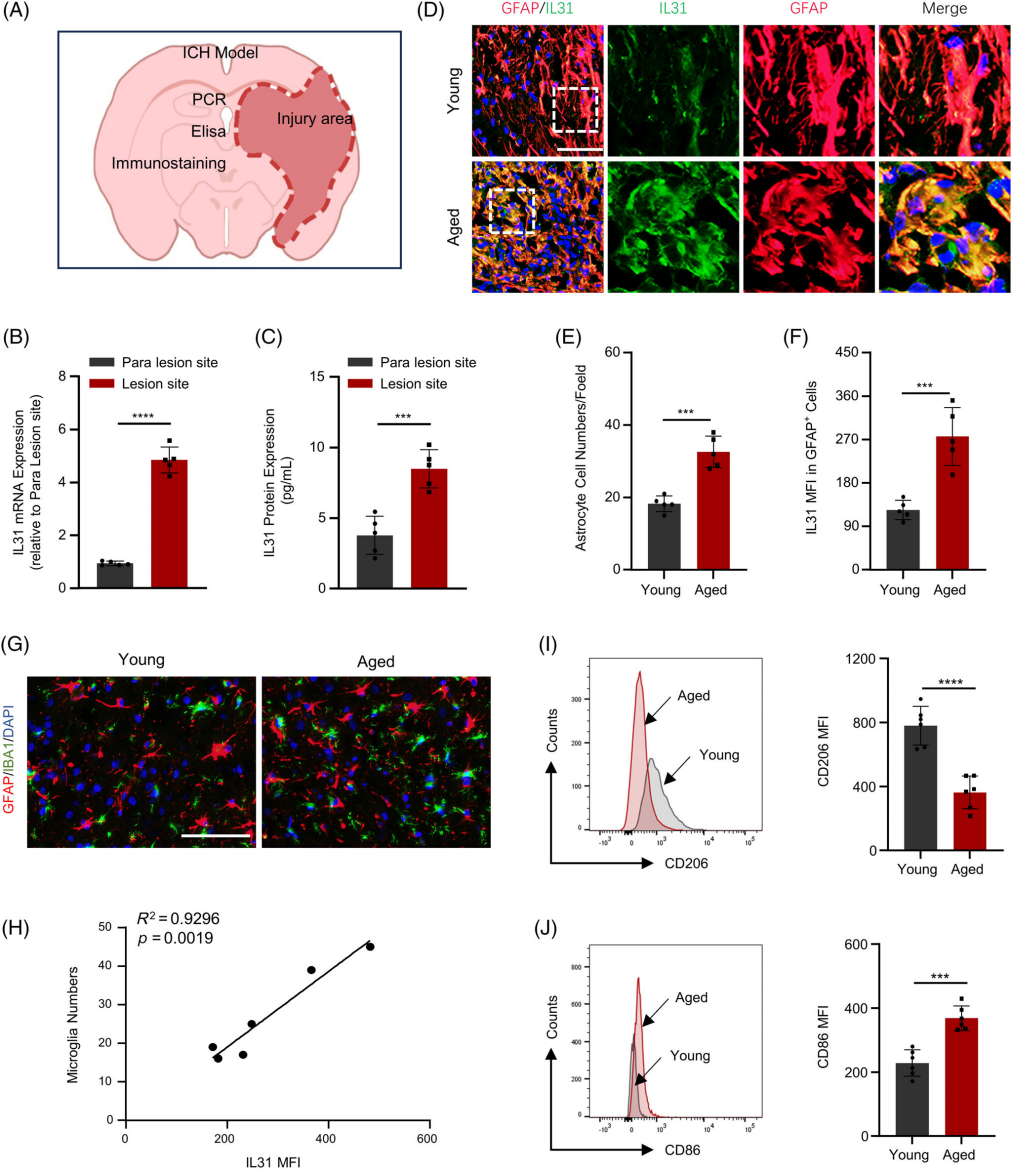
Elderly mice express higher IL31 and cause spread of inflammation after intracerebral hemorrhage (ICH). (A) After constructing mouse autologous blood ICH model. (B) polymerase chain reaction (PCR) to detect the expression of IL31 in the hematoma area and paraneatoma area of mice(*n* = 3). (C) Western Blot to detect the expression of IL31 in the hematoma area and paraneatoma area of mice(*n* = 3). (D–F) Immunofluorescence to detect and statistically analyze the expression of GFAP and IL31 in the hematoma area of elderly and young mice (*n* = 3). (G) Immunofluorescence to detect the composition of astrocytes and microglia in the hematoma area of elderly and young mice. (H) Regression analysis of microglia and IL31. (I) Flow detection of CD206 expression in the hematoma area of elderly and young mice (*n* = 3). (J) Flow detection of CD86 expression in the hematoma area of elderly and young mice (*n* = 3; *p < 0.05; **p < 0.01; ***p < 0.001; ****p < 0.0001).

To delve deeper into the role of IL31 following ICH across different age groups, 4‐month‐old (representing young patients) and 18‐month‐old (representing elderly patients) mice were used to establish an autologous blood–brain hemorrhage model (Figure 2A). Initially, IL31 expression variations were assessed in distinct regions using PCR assay (Figure 2B) and western blot (Figure 2C). The findings revealed a significant surge in IL31 expression within the injury area. Concurrently, the results of immunofluorescence in brain tissue sections mirrored those observed in humans: astrocytes exhibited marked activation in elderly mice post‐ICH, coupled with a corresponding increase in IL31 expression (Figure 2D–F). Given the similarity between the C57BL/6 mouse ICH model and human cases, the B6 mouse ICH model was selected for both phenomenological and mechanistic investigations.

The activation of astrocytes and microglia triggers heightened neuroinflammation. The immunofluorescence assay was performed to scrutinize the expression of GFAP and IBA1 after ICH in both old and young mice. The findings indicated an upswing in astrocytes and microglia in the injury area of elderly mice, and regression analysis unveiled a positive correlation between microglia numbers and IL31 expression (Figure 2G,H). This implies that as activated astrocytes released more IL31, microglia were drawn to recruit. Consequently, microglia activation led to further propagation of neuroinflammation. This study also assessed the expression of inflammatory cytokines in the injury area using flow cytometry: anti‐inflammatory makers such as CD206 and IL4, primarily responsible for neuroprotection, exhibited an increase in young ICH mice (Figures 2I and S2A). In contrast, the expression of inflammatory makers such as CD86 and IL1β markedly surged in elderly ICH mice (Figures 2J and S2B). Furthermore, TUNEL assay revealed a significant rise in apoptotic cells among elderly individuals, with a positive correlation observed between apoptotic cells and IL31 (Figure S2C–E). These results indicate that the activation of astrocytes post‐ICH in elderly mice results in a substantial release of IL31, leading to the recruitment of a large number of microglia, thereby propagating inflammation and ultimately causing apoptosis.

### 3.3 Increased IL31 leads to neuronal apoptosis and further microglia activation

**FIGURE 3:**
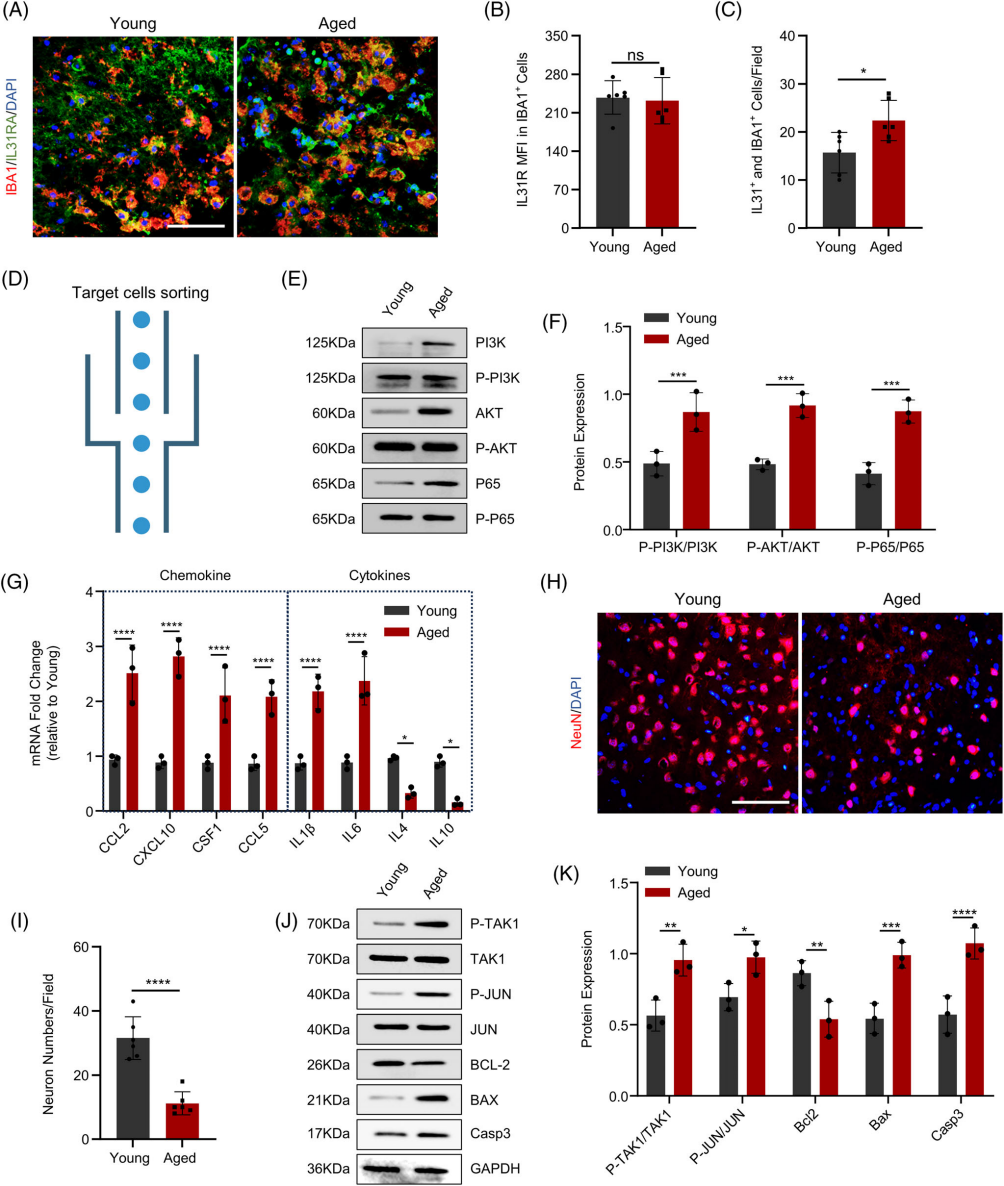
Increased IL31 leads to neuronal apoptosis and further microglia activation. (A–C) Immunofluorescence to detect the expression of IL31R‐positive microglia in the hematoma area of elderly and young mice after intracerebral hemorrhage (*n* = 3). (D) Cell sorting pattern. (E,F) Western blot to detect the expression of PI3K‐related pathway molecules in the hematoma area of elderly and young mice (*n* = 3). (G) Polymerase chain reaction to detect the expression of inflammatory factors and chemokines in the hematoma area of old and young mice (*n* = 3). (H,I) immunofluorescence to detection of neuronal expression in the hematoma region of elderly and young mice (*n* = 3). (J,K) Western blot detection of apoptosis‐related pathway molecules in the hematoma region of elderly and young mice (*n* = 3; *p < 0.05; **p < 0.01; ***p < 0.001; ****p < 0.0001).

After establishing the differential expression of IL31 following ICH in mice of varying ages, the expression of IL31R in microglia was detected using an immunofluorescence assay. Surprisingly, no significant difference was observed in IL31R expression between elderly and young microglia (Figure 3A,B). However, it was found the proportion of IL31R‐positive microglia in the brain of elderly ICH mice was significantly higher than that of young ICH mice (Figure 3C). Armed with the clear presence of the IL31 receptor, neurons and microglia were isolated through cell sorting (Figure 3D) and examined their downstream pathways post‐stimulation with IL31. A significantly heightened expression of inflammatory activation pathways, such as P‐PI3K, P‐Akt, and P‐P65, was observed in microglia following IL31 stimulation in elderly individuals (Figure 3E,F). Additionally, chemokines alongside toxic inflammatory factors such as Il1β and IL6 were markedly elevated in elderly individuals (Figure 3G). Notably, the number of neurons in elderly individuals was notably fewer than that in their young counterparts (Figure 3H,I), with downstream pathway assays revealing an increased expression in apoptosis‐related pathway molecules (Figure 3J,K). These findings suggest that the substantial release of IL31 from astrocytes post‐ICH in elderly mice promotes heightened inflammation in microglia and subsequent neuronal apoptosis, ultimately contributing to a less favorable prognosis. For this purpose, we used inhibitors of the PI3K‐AKT signaling pathway and TAK‐1 signaling pathway in ICH mice. We found that inhibition of the PI3K and TAK‐1 pathways effectively improved the prognosis of ICH mice, as evidenced by the maintenance of neuron numbers, improvement in behavioral scores, and down‐regulation of inflammatory cytokines (Figure S3).

### 3.4 Inhibition of IL31 expression promotes recovery of neurological function after ICH

**FIGURE 4:**
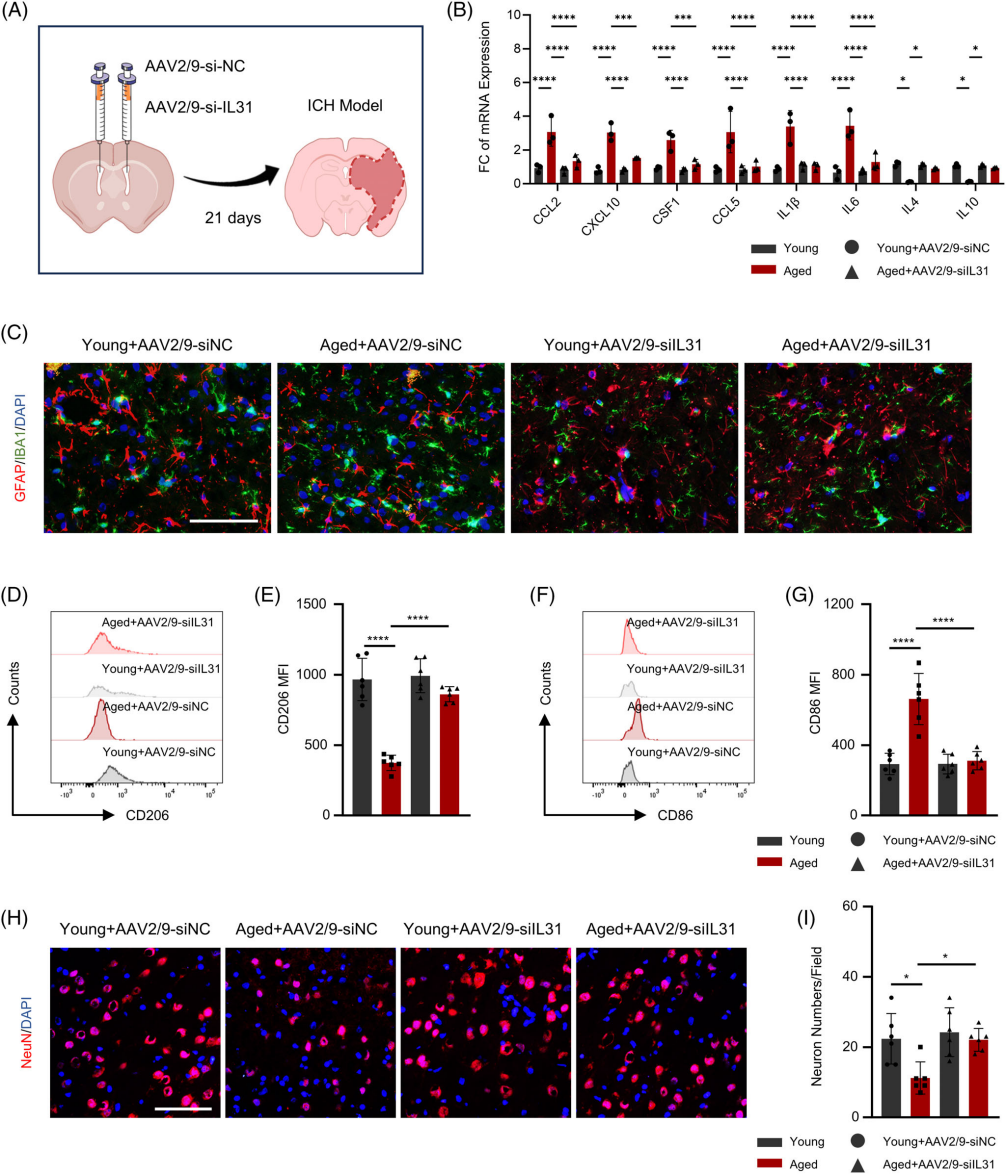
Neuroinflammation in intracerebral hemorrhage (ICH) can be reduced after interfering with IL31 expression. (A) The construction process of mouse model: control or IL31 interfering virus was injected into the lateral ventricle of mice first, and then the ICH model was constructed after 21 days. (B) Polymerase chain reaction detected the changes in the expression of inflammatory factors and chemokines before and after interfering with IL31 in elderly and young mice (*n* = 3). (C) Immunofluorescence detected the expression of astrocytes and microglial cells before and after interfering with IL31 in elderly and young mice. (D,E) Flow detection of CD206 expression before and after interference with IL31 in elderly and young mice (*n* = 3). (F,G) Flow detection of CD86 expression before and after interference with IL31 in elderly and young mice. (H,I) Immunofluorescence detection of neuron numbers before and after interference with IL31 in elderly and young mice (*n* = 3; *p < 0.05; **p < 0.01; ***p < 0.001; ****p < 0.0001).

To further investigate the role of IL31 on ICH, both AAV2/9‐si‐NC and AAV2/9‐si‐IL31 viruses were administered into the lateral ventricles of both young and elderly mice, subsequently evaluating their infection efficiency (Figure S4A,B). Following a 21‐day postoperative period, ICH modeling was re‐initiated (Figure 4A). Thereafter, a PCR assay was performed to detect the expression of chemokines and inflammatory factors, revealing that interference with IL31 led to a notable reduction in the expression of chemokines and pro‐inflammatory cytokines, particularly evident in the elderly mice. Concurrently, there was an upward trend in the expression of anti‐inflammatory cytokines (Figure 4B). Immunofluorescence assessments of astrocytes and microglia yielded corroborating results: the quantity of both astrocytes and microglia significantly decreased, with a more pronounced effect observed in the elderly mice (Figures 4C and S4C). Subsequent flow cytometric assays for CD206 and CD86 demonstrated an increase in CD206 expression and a decrease in CD86 expression post‐IL31 interference (Figure 4D–G). Moreover, after IL31 interference, there was a substantial reduction in the number of apoptotic cells (Figure S4D,E).

**FIGURE 5:**
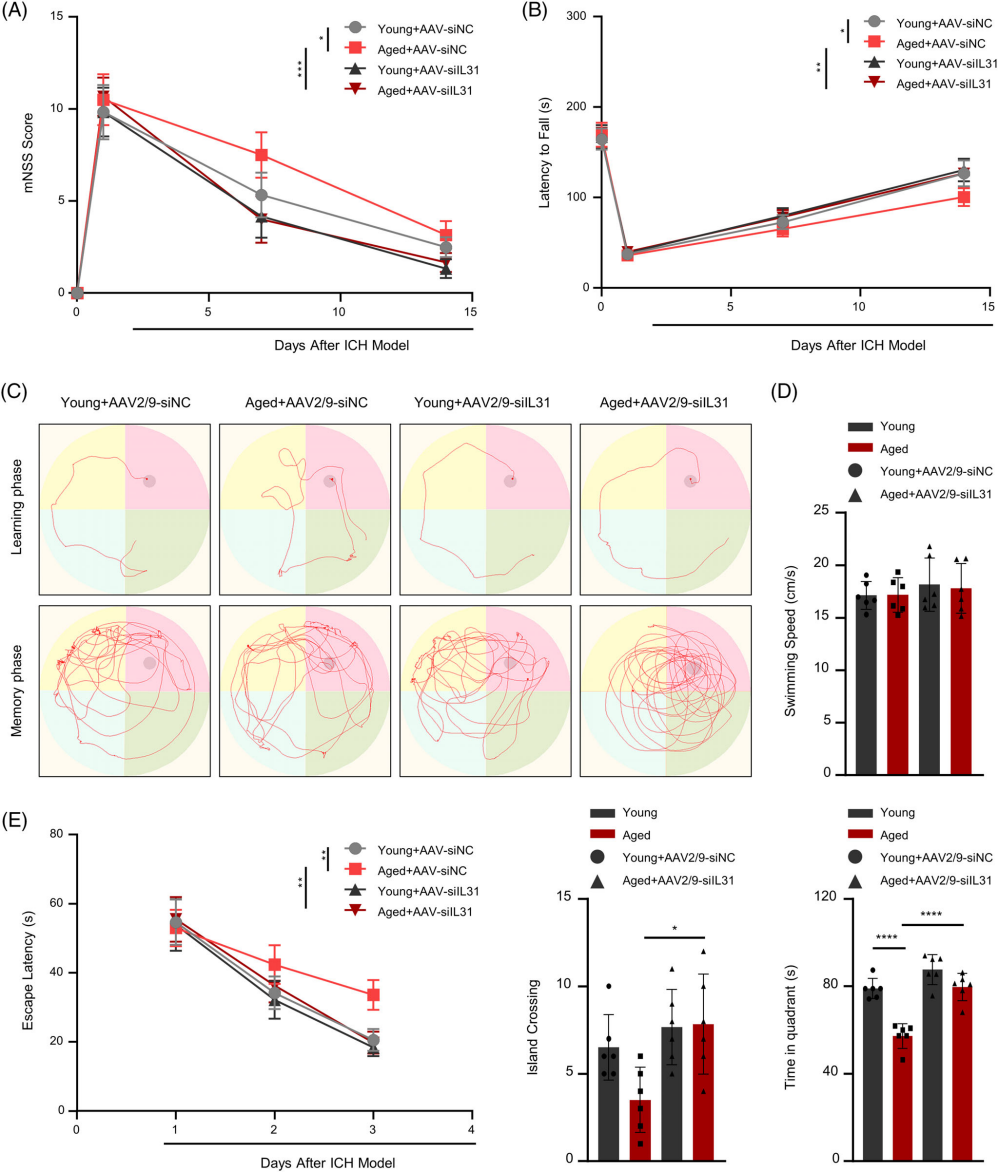
Interfering with IL31 can promote the recovery of neurological functions. Interfering with IL31 can promote the recovery of neurological functions. (A) modified neurological severity score (mNss) scores before and after interference with IL31 in elderly and young mice (*n* = 3). (B) Rotarod detection of motor function changes before and after interference with IL31 in elderly and young mice (*n* = 3). (C) Characteristic performance graphs of the motor water maze before and after interference with IL31 in elderly and young mice. (D) Statistics of motor swimming speed, number of times of crossing the island, and residence time in the target quadrant before and after interference with IL31 in elderly and young mice (*n* = 3). (E) The difference of delayed period of escaping from the pool during learning period before and after interference with IL31 (*n* = 3; *p < 0.05; **p < 0.01; ***p < 0.001; ****p < 0.0001).

Following the evaluation of changes in inflammatory factors and cellular function after viral interference with IL31, the recovery of neurological function in mice across various age groups was assessed. The mNSS score, a crucial metric for evaluating a murine neurological function, showed a marked improvement after IL31 interference, particularly evident in the elderly mice (Figure 5A). Additionally, results from the rotarod test supported our hypothesis that suppressing IL31 protects the neurological function of mice post‐ICH, with the elderly mouse exhibiting enhanced and expedited recovery (Figure 5B). With Morris water maze test, the swimming speed of mice remained unchanged after IL31 interference, regardless of age. However, a substantial increase was observed in island crossings and target quadrant dwell time, with a much higher percentage of increase observed in the elderly mice than in young mice (Figure 5C,D). Furthermore, in terms of the delayed escape from the pool during the learning phase, elderly mice demonstrated greater benefit after IL31 interference (Figure 5E). These outcomes suggest that IL31 interference leads to improved neurological recovery in ICH mice, with elderly mice experiencing greater advantages.

**FIGURE 6:**
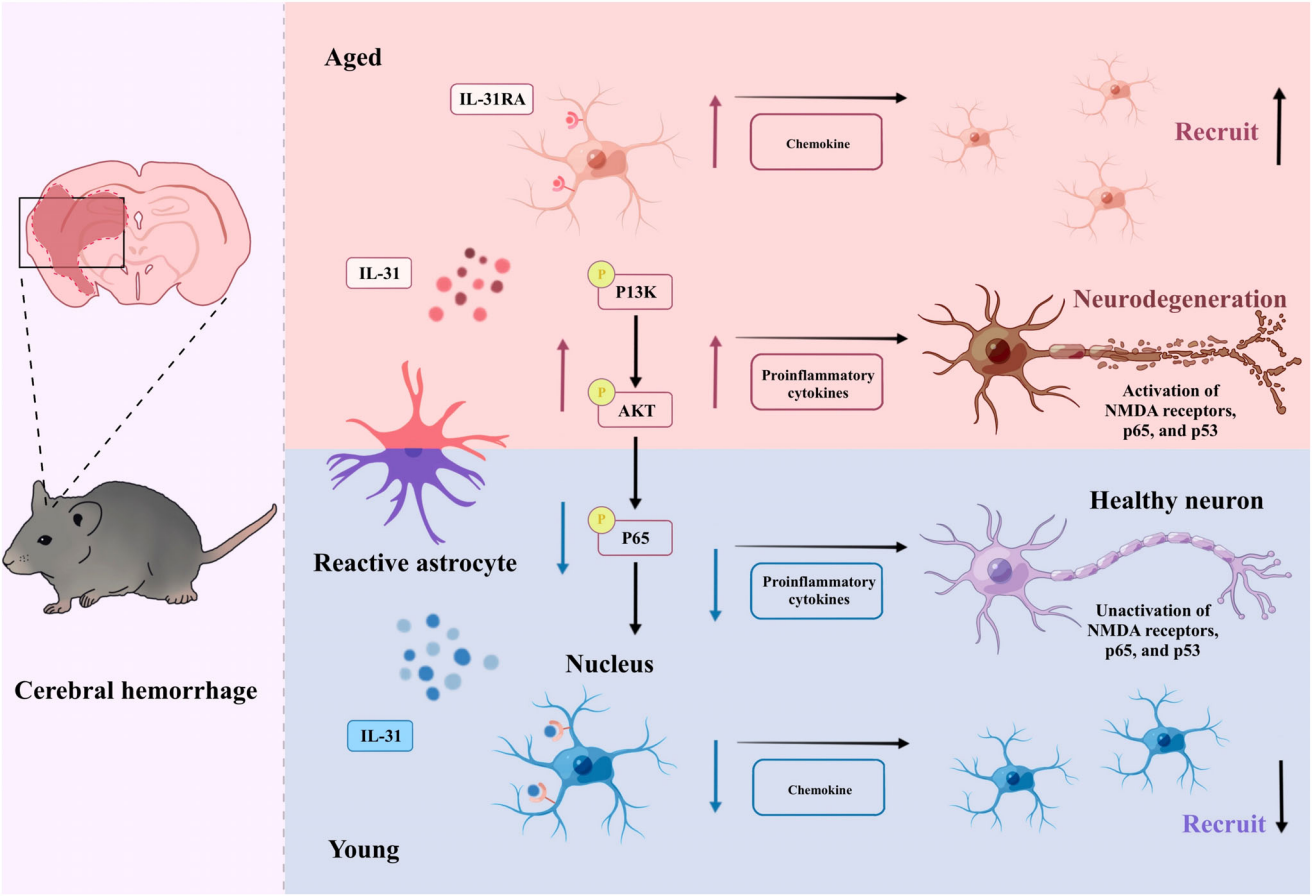
High concentrations of astrocyte‐derived IL31 in the central nervous system (CNS) immune microenvironment of elderly intracerebral hemorrhage (ICH) patients lead to neurological deficits and poor prognosis. The massive activation of astrocytes after ICH in elderly mice releases IL31, which binds to microglia indicating that IL31R binds to each other causing massive chemotaxis of microglia. Subsequent activation of NMDA receptors, P65 and P53, and so forth, causes degenerative changes in neurons, resulting in neuronal apoptosis and leading to a malignant outcome. In contrast, young mice are more inhibited NMDA receptor, P65 and P53, and so forth, which reduces neuronal apoptosis and thus promotes the recovery of neurological function.

This study demonstrated that IL31 may play a pivotal role in shaping the neural microenvironment and influencing prognosis post‐ICH in mice across various age groups. The results revealed extensive activation of astrocytes in elderly mice following ICH, resulting in the release of IL31, which, in turn, interacted with IL31R on microglial surfaces, culminating in a substantial inflammation response. This was succeeded by the activation of NMDA receptors, P65, and P53, initiating degenerative alterations in neurons, ultimately leading to apoptosis and a malignant outcome. Conversely, young mice displayed a more restrained response in NMDA receptors, P65, and P53, thereby reducing neuronal apoptosis and consequently fostering neurological function recovery (Figure 6).

## 4 DISCUSSION

ICH is a devastating cerebrovascular disease that induces both primary and secondary damage [22]. The initial injury triggers a unique neuroimmune microenvironment, propagating inflammation and prompting extensive cellular apoptosis, culminating in irreversible harm [23, 24, 25]. Ongoing research primarily targets inflammation post‐ICH, aiming to mitigate the impacts stemming from microglia hyperactivation [26, 27]. Consequently, inhibiting the excessive secretion of pro‐inflammatory mediators and overactive microglia emerges as a pivotal approach for treating ICH and enhancing prognosis. The investigations in this study delve into modifications within the neural microenvironment post‐ICH, particularly scrutinizing the factors contributing to the graver prognosis in elderly ICH cases [28, 29]. Initially, the findings were validated using intraoperatively collected patients' tissues. Subsequently, an ICH model was established employing both elderly and youth mice. This facilitated an exploration of the IL31 mechanism in the context of aging after ICH. The observations in this study initially revealed a significant surge in astrocyte‐released IL31 after ICH, with the spike being notably prominent in elderly individuals. Subsequently, heightened IL31 expression could further activate microglia and trigger neuroinflammtion and neuronal apoptosis through PI3K and related pathways, a phenomenon more conspicuous in elderly mice. Ultimately, inhibiting IL31 expression demonstrated a reduction in neuroinflammation, fostering neurological function recovery, with more pronounced benefits observed in elderly mice.

Secondary brain damage ensuing from ICH stems from a series of pathological processes, including oxidative stress, mitochondrial dysfunction, prolonged inflammation, and central immune response [30]. The decline in neurological function following ICH is attributable partially to neuroinflammation, a phenomenon particularly pronounced among the elderly. An early and appropriate inflammatory response proves instrumental in injury repair and facilitates neuronal remodeling. However, an excessively aggressive inflammatory reaction can result in enduring neuronal impairment. Persistent inflammation prompts the release of chemokines from injured neural tissue, attracting peripheral immune cells to the injury site. Simultaneously, microglia swiftly activate, generating copious amounts of inflammatory mediators, thereby exacerbating neuroinflammation. Hence, arresting the escalation of inflammation in its early stages remains a primary focus in ongoing research.

With the increasing focus on neuroinflammation post‐ICH, we have uncovered distinct roles of astrocytes and microglia within different microenvironments. They release various inflammatory factors and chemokines that significantly influence the activity of neurons, oligodendrocytes, and other cells. This, in turn, affects axon extension and leads to uncontrolled immune responses, ultimately exacerbating ICH‐related neurological damage. Given that the body's immune system dictates the onset and progression of various types of inflammation, excessive immune reactions following brain injury can incite unmanageable neuroinflammatory responses and secondary damage. Consequently, investigating neuroinflammation in the aftermath of ICH, as well as understanding the regulation of glial cells in areas of infiltrating injury, constitutes a pivotal area of ICH research. This study revealed that by modulating the expression of IL31, the activation of astrocytes and microglia in the affected region markedly diminished. This, in turn, affected the secretion of harmful inflammatory factors and diverse chemokines, leading to an improvement in neurological function recovery. These shifts were particularly pronounced in elderly individuals, indicating heightened IL31 expression in the elderly, resulting in more severe outcomes.

While the intricate molecular mechanisms through which IL31 exerts its detrimental effects on ICH are multifaceted, the present study suggests that IL31 modulates the inflammatory response of microglia through the PI3K‐AKT‐related signaling pathway and influences neuronal apoptosis through the TAK1‐related signaling pathway. This underscores the importance of investigating the specific biological functions of IL31 post‐ICH, especially in elderly patients. Through this study, we aim to bridge existing research gaps in this domain. Additionally, the development of monoclonal antibodies targeting this pathway holds promise for facilitating the transition from laboratory findings to clinical applications, offering substantial clinical therapeutic value and socioeconomic benefits.

## 5 CONCLUSION

In this study, we confirmed that astrocyte‐derived IL31 contributes to the poor prognosis of patients with ICH. The higher capacity of astrocytes to secrete IL31 in elderly ICH patients may be a major contributor to the poor prognosis of elderly ICH patients. This is the first study to elucidate the role played by IL31 in the pathologic process of ICH, and targeting IL31 inhibition may contribute to the recovery of ICH patients, especially elderly ICH patients. This would provide a new approach to clinical strategies for targeting astrocytes in early ICH management.

## AUTHOR CONTRIBUTIONS

*Conceptualization*: WJT, YY, KJK, and PPG. *Methodology*: RJ and ZCL. *Validation*: ZCL and CXW. *Formal analysis*: QY and JBS. *Writing—original draft preparation*: RJ and ZCL. *Writing—review and editing*: YY, KJK, and PPG. *Visualization*: CXW. *Supervision*: YY, KJK, and PPG. All authors have read and agreed to the published version of the article.

## FUNDING INFORMATION

This research was funded by the National Natural Science Foundation of China (82271415), Postgraduate Research and Practice Innovation Program of Jiangsu Province (KYCX22‐3364), and the Nantong Natural Science Foundation (JC12022063).

## CONFLICT OF INTEREST STATEMENT

The authors declare no conflicts of interest.

## ETHICS STATEMENT

All human tissue specimens and animal experiments were conducted with the approval of the Ethics Committee of Nantong University (2022‐K004 and S20180816‐012).

## Supporting information


**Data S1:** Supporting information.

## Data Availability

Data are available on reasonable request.
