# Supplementary material for: Astrocyte‐derived Interleukin‐31 causes poor prognosis in elderly patients with intracerebral hemorrhage
Source: Brain Pathol. 2024 Feb 14;34(5):e13245. doi: 10.1111/bpa.13245 (PMC11328350; doi:10.1111/bpa.13245)
Supplement: Supplementary file 1 — Data S1: Supporting information. [file BPA-34-e13245-s001.docx]

**Supplementary materials for**

**Astrocyte-derived IL31 causes poor prognosis in elderly patients with intracerebral hemorrhage**

Rui Jiang^1,2, 3, #^, Zhichao Lu^1,4, #^, Chenxing Wang,^1,4, #^, WenJun Tu^9^, Qi Yao^1,2, 3^, Jiabing Shen^5^, Xingjia Zhu^1,4^, Ziheng Wang^1,8^, Yixun Chen^4^, Yang Yang^6*^, Kaijiang Kang^10*^, PeiPei Gong^1,2,3*^

^1^ Department of Neurosurgery, Affiliated Hospital of Nantong University, Medical School of Nantong University, Nantong, Jiangsu, 226001, China.

^2^ Neuro-Microscopy and Minimally Invasive Translational Medicine Innovation Center, Affiliated Hospital of Nantong University, Nantong, Jiangsu, 226001, China.

^3^ Jiangsu Medical Innovation Centre, Neurological disease diagnosis and treatment center, Affiliated Hospital of Nantong University, Nantong, Jiangsu, 226001, China.

^4^ Research Center of Clinical Medicine, Affiliated Hospital of Nantong University, Nantong, Jiangsu, 226001, China.

^5^ Department of Neurology, Affiliated Hospital of Nantong University, Affiliated Hospital of Nantong University, Nantong, Jiangsu, 226001, China.

^6^ Department of Physiology, School of Medicine, Nantong University, Nantong, Jiangsu 226001, China.

^7^ Department of Trauma Center, Affiliated Hospital of Nantong University, Medical school of

Nantong University, Nantong, 226001, China.

^8^ Centre for Precision Medicine Research and Training, Faculty of Health Sciences, University of Macau.

^9^ Department of Neurology, Beijing Tiantan Hospital, Capital Medical University; China National Clinical Research Center for Neurological Diseases, Beijing, 100070, China.

^10^ Department of Neurology, Beijing Tiantan Hospital, Capital Medical University, Beijing, China.

# These authors contributed equally

* Corresponding authors

Peipei Gong : ntgpp@ntu.edu.cn

Kaijiang Kang : kangkaijiang678@126.com

Yang Yang : yangyang286228@ntu.edu.cn

**Supplementary Figures**

**
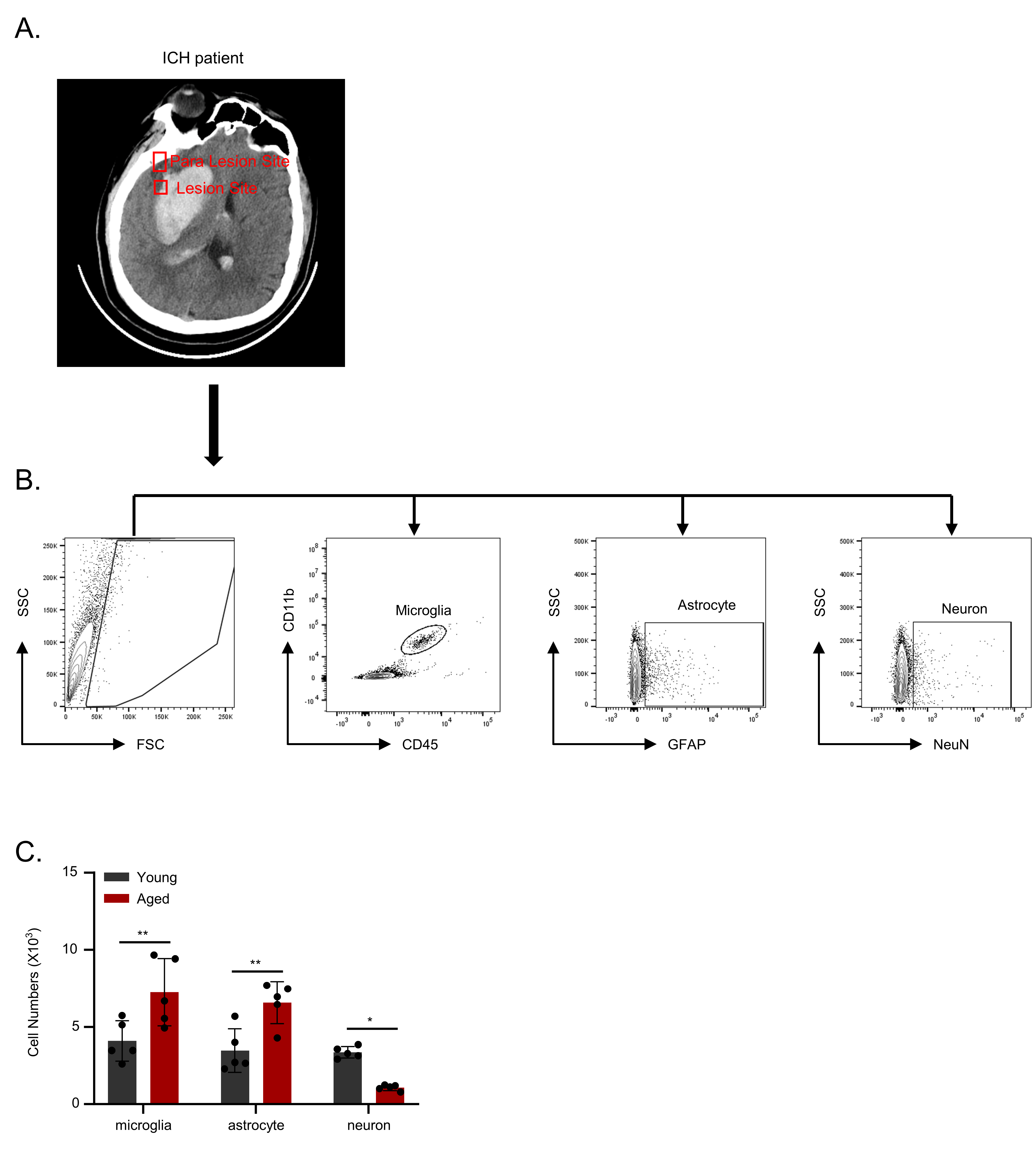
**

**Supplementary Figure. 1: Sampling sites for flow cytometry and detection strategies.**

A: Schematic representation of the tissue collected from patients undergoing ICH surgery: along the cortical fistula opening into the hematoma cavity, the part close to the hematoma is the hematoma zone, and the part close to the cortex is the paraneoplastic zone; B-C: Flow cytometry assay for the proportion of astrocytes, microglia, and neurons in the hematoma zones of older and younger patients (n=5).


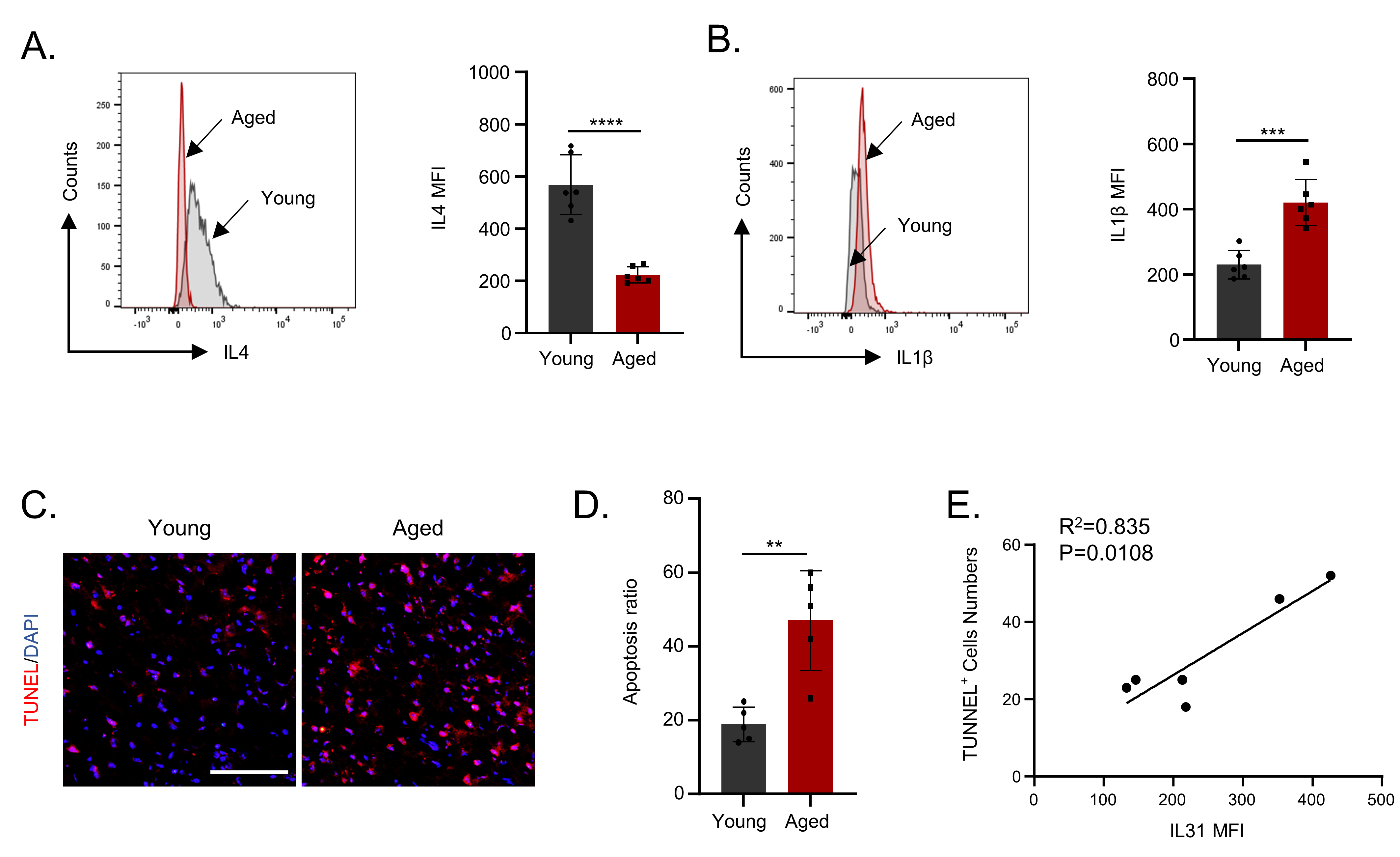


**Supplementary Figure. 2: Elderly ICH mice showed higher levels of inflammatory response and higher proportions of apoptosis in the injury zone.**

A: Flow cytometry assay for IL4 expression detection in the hematoma area of old and young mice (n=3); B: Flow cytometry assay for IL1β expression detection in the hematoma area of old and young mice (n=3); C-D: TUNNEL assay for the apoptosis of peripheral neurons in the hematoma area of old and young mice; E: Regression analysis of the number of apoptotic cells and IL31 expression (n=3).


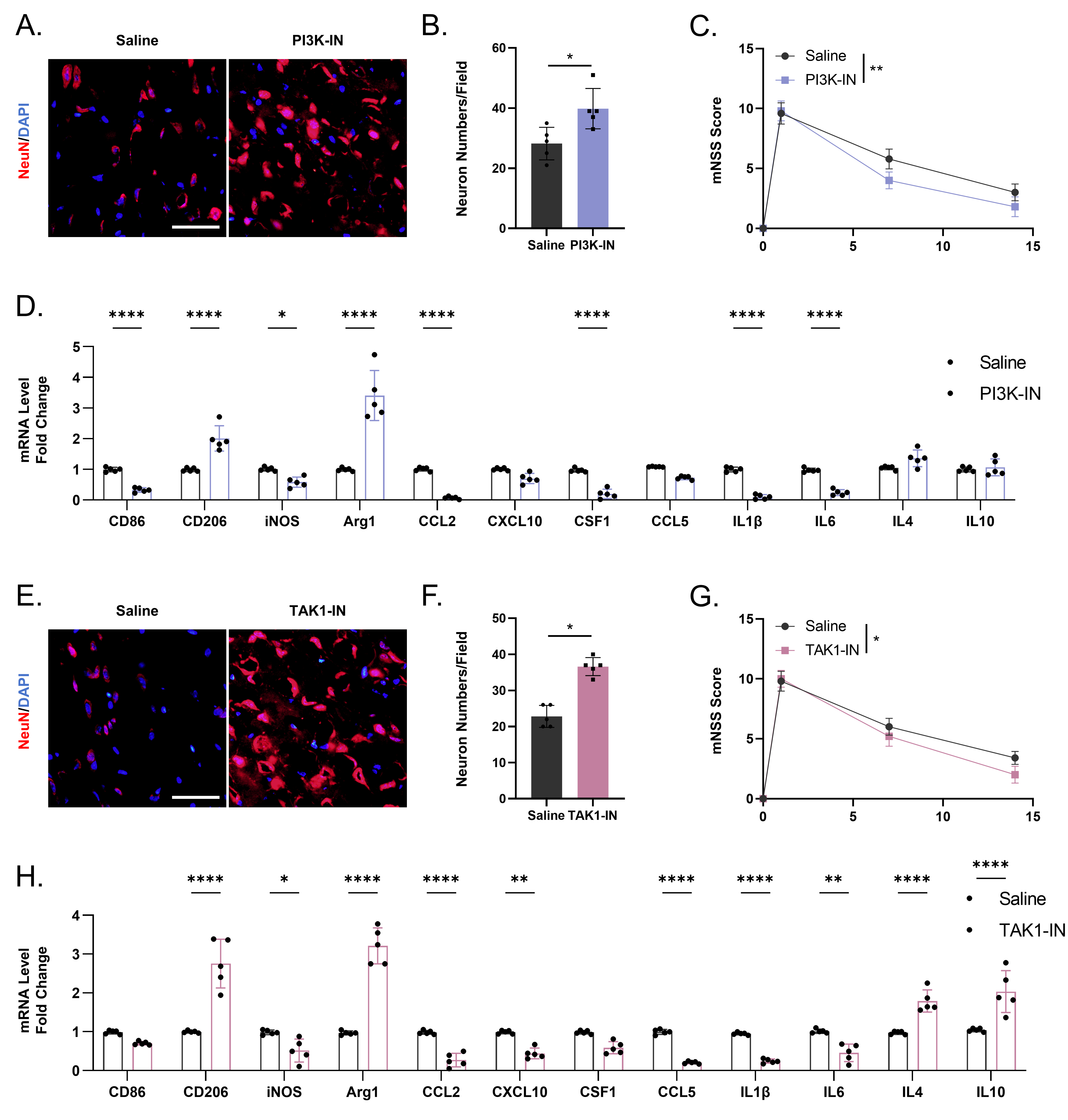


**Supplementary Figure. 3: Inhibition of the PI3K/AKT and TAK-1 Pathways Inhibits Recovery of Neural Function**

A-B：The identification of neuronal expression within the affected region and quantification of neurons through immunofluorescence subsequent to the administration of PI3k inhibitors(n=3);C: The mNss scores of the control and inhibitor groups were assessed(n=3);D: PCR detection was used to assess the expression of different inflammatory factors and chemokines in both the control group and the group treated with a PI3K inhibitor(n=3);E-F: The identification of neuronal expression within the affected region and quantification of neurons through immunofluorescence subsequent to the administration of TAK-1 inhibitors(n=3);G: The mNss scores of the control and inhibitor groups were assessed(n=3);H: PCR detection was used to assess the expression of different inflammatory factors and chemokines in both the control group and the group treated with TAK-1 inhibitor(n=3).


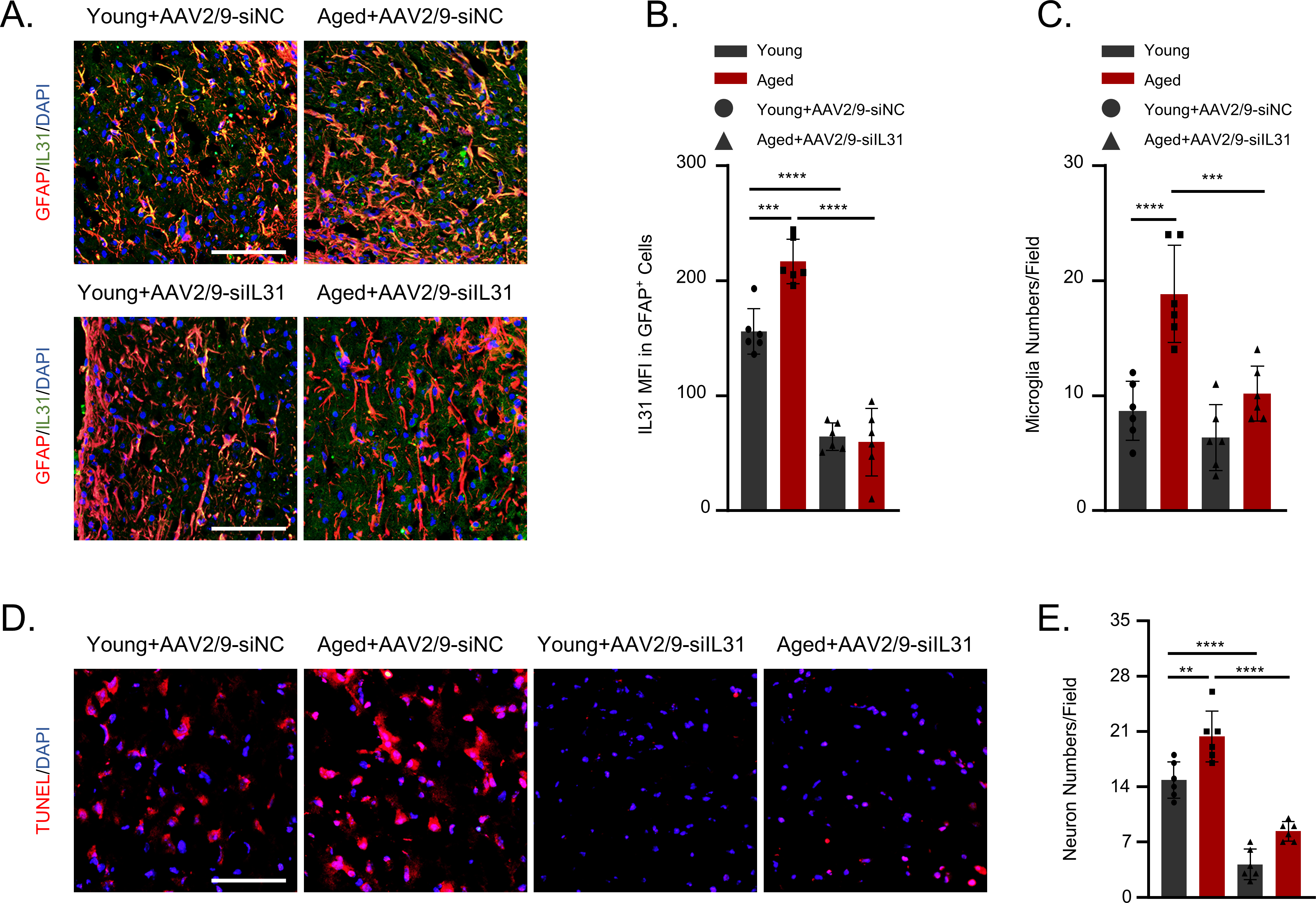


**Supplementary Figure. 4:** **Knockdown of IL31 inhibited microglia responses and reduced the percentage of apoptosis in the lesion zone after ICH.**

A-B: Immunofluorescence assay to detect the expression of astrocytes and IL31 before and after interference with IL31 in old and young mice (n=3); C: Expression of microglia before and after interference with IL31 in old and young mice (n=3); D-E: TUNNEL assay to detect neuronal apoptosis before and after interference with IL31 in old and young mice (n=3).

**Supplementary Tables**

**Supplementary Table 1. Sequence of primer pairs for the target genes.**

| Gene | Forward primer (5′-3′) | Reverse primer (5′-3′) |
| --- | --- | --- |
| IL31(human) | CGTCCGTTTACTACGACCAAGTG | GGCAGCGTGTAATTCTGGGACA |
| IL31(mouse) | GGGCCTTTGATCTGCTTCCT | TCCAGTCCCTACAGGGTAGC |
| CCl2 | GCTACAAGAGGATCACCAGCAG | GTCTGGACCCATTCCTTCTTGG |
| CXCL10 | ATCATCCCTGCGAGCCTATCCT | GACCTTTTTTGGCTAAACGCTTTC |
| CSF1 | GCCTCCTGTTCTACAAGTGGAAG | ACTGGCAGTTCCACCTGTCTGT |
| CCL5 | CCTGCTGCTTTGCCTACCTCTC | ACACACTTGGCGGTTCCTTCGA |
| IL1β | TGGACCTTCCAGGATGAGGACA | GTTCATCTCGGAGCCTGTAGTG |
| IL6 | AGACAGCCACTCACCTCTTCAG | TTCTGCCAGTGCCTCTTTGCTG |
| IL4 | ATCATCGGCATTTTGAACGAGGTC | ACCTTGGAAGCCCTACAGACGA |
| IL10 | CGGGAAGACAATAACTGCACCC | CGGTTAGCAGTATGTTGTCCAGC |
| GAPDH | ATGACCACAGTCCATGCCATC | GAGCTTCCCGTTCAGCTCTG |

**Supplementary Table 2. Antibodies used in this study.**

| Reagents | Company | Cat No. | application |
| --- | --- | --- | --- |
| Rabbit anti- GFAP | Abcam | ab7260 | Immunofluorescence staining |
| Mouse anti- GFAP | Abcam | ab279290 | Immunofluorescence staining |
| Rabbit anti- IBA1 | Abcam | ab178846 | Immunofluorescence staining |
| Mouse anti- IBA1 | Abcam | ab283319 | Immunofluorescence staining |
| Rabbit anti-IL31 | Abcam | ab62579 | Immunofluorescence staining |
| Rabbit anti-IL31R | Abcam | ab113498 | Immunofluorescence staining |
| Mouse anti-NeuN | Abcam | ab104224 | Immunofluorescence staining |
| Mouse anti-PI3K | Abcam | ab302958 | Western blot |
| Rabbit anti-P-PI3K | Abcam | ab127617 | Western blot |
| Rabbit anti-P65 | Abcam | ab109458 | Western blot |
| mouse anti P-AKT | Abcam | ab283852 | Western blot |
| Rabbit anti- TAK1 | Abcam | ab109526 | Western blot |
| Rabbit anti- P-TAK1 | Abcam | ab109404 | Western blot |
| Rabbit anti- P-JUN | Abcam | ab32385 | Western blot |
| Rabbit anti- JUN | Abcam | ab40766 | Western blot |
| Rabbit anti- BCL-2 | Abcam | ab182858 | Western blot |
| Rabbit anti- BAX | Abcam | ab32503 | Western blot |
| Rabbit anti- Caspase3 | Abcam | ab13847 | Western blot |
| Alexa Fluor® 488 donkey anti-Mouse IgG | Abcam | ab150113 | secondary antibody |
| Alexa Fluor® 488 donkey anti-Rabbit IgG | Abcam | ab150077 | secondary antibody |
| Alexa Fluor® 594 donkey anti-Mouse IgG | Abcam | ab150116 | secondary antibody |
| Alexa Fluor® 594 donkey anti-Rabbit IgG | Abcam | ab150080 | secondary antibody |
| HRP-conjugated Affinipure Goat Anti-Rabbit IgG | Proteintech | SA00001-2 | secondary antibody |
| HRP-conjugated Affinipure Goat Anti-Mouse IgG | Proteintech | SA00001-1 | secondary antibody |
| CD45 Monoclonal Antibody (HI30), Brilliant Violet™ 421, eBioscience™ | ThermoFisher | 404-0459-42 | Flow cytometry |
| CD11b Monoclonal Antibody (M1/70), Brilliant Violet™ 650, eBioscience™ | ThermoFisher | 416-0112-82 | Flow cytometry |
| NeuN Antibody, anti-human/mouse/rat, PE | Miltenyi | 130-119-493 | Flow cytometry |
| GFAP Monoclonal Antibody (GA5), Alexa Fluor™ 488, eBioscience™ | ThermoFisher | 53-9892-82 | Flow cytometry |
| IL-31 Monoclonal Antibody (31SNEZE), eFluor™ 660, eBioscience™ | ThermoFisher | 50-9319-42 | Flow cytometry |
| CD45 Monoclonal Antibody (30-F11), PE, eBioscience™ | ThermoFisher | 12-0451-82 | Flow cytometry |
| CD11b Monoclonal Antibody (M1/70), Alexa Fluor™ 488, eBioscience™ | ThermoFisher | 53-0112-82 | Flow cytometry |
| CD86 (B7-2) Monoclonal Antibody (GL1), PE-Cyanine5, eBioscience™ | ThermoFisher | 15-0862-82 | Flow cytometry |
| CD206 (MMR) Monoclonal Antibody (MR6F3), APC, eBioscience™ | ThermoFisher | 17-2061-82 | Flow cytometry |

**Supplementary Table 3. Subjects’ characteristics.**

|  | Young ICH | Elderly ICH |
| --- | --- | --- |
| Cohort size | 5 | 5 |
| Age (year) | 24-45 | 65-81 |
| Sex (male), n (%) | 3(60) | 3(60) |
| Surgery within 1 day of ICH, n (%) | 4(80) | 3(60) |
| Surgery within 2 day of ICH, n (%) | 0(0) | 1(20) |
| Surgery within 3 day of ICH, n (%) | 1(20) | 1(20) |
| In hospital mortality, n (%) | 0(0) | 0(0) |
| Length of hospitalization (days), Median (IQR) | 11(7-15) | 13.5(9-19) |
| GCS at discharge (survivors), Median  (IQR) | 14(13-15) | 13.5 (11–15) |
